# Three‐dimensional Numerical Simulation of Gas‐particulate Flow around Breathing Human and Particulate Inhalation

**DOI:** 10.1063/1.2204538

**Published:** 2006-05-05

**Authors:** Yasuhiro Shimazaki, Masaaki Okubo, Toshiaki Yamamoto

**Affiliations:** Department of Mechanical Engineering, Osaka Prefecture University, 1‐1 Gakuen‐cho, Sakai, Osaka 599‐8531, JAPAN; Tohoku University; Tohoku University

## Abstract

It is important to predict the environment around the breathing human because inhalation of virus (avian influenza, SARS) is recently severe worldwide problem, and air pollution caused by diesel emission particle (DEP) and asbestos attract a great deal of attention. In the present study, three‐dimensional numerical simulation was carried out to predict unsteady flows around a breathing human and how suspended particulate matter (SPM, diameter∼1 μm) reaches the human nose in inhalation and exhalation. In the calculation, we find out smaller breathing angle and the closer distance between the human nose and pollutant region are effective in the inhalation of SPM.

## INTRODUCTION

In recent years, decline of air quality becomes one of significant problems and people from all over the world are concerned as human safety [1]. There are two main types of air pollutant: particulate pollutant and gas pollutant. Damages by SARS [2], avian influenza [3], diesel emission particles [4], and asbestos [5] are the good examples of particulate pollutant. Many researches have been performed to deal with the increasing problems of such air pollutants [6], [7]. However, few studies have been performed to address the theme of human with unsteady breathing [8], [9]. Two-dimensional numerical simulation was carried out, and the time-dependent pollutant concentrations were determined until this point [10]. In this paper, calculating area was advanced to three-dimension, real particle with size and mass. In the simulation, the breathing process alternated between inhalation and exhalation as the unsteady breathing at nose.

## BASIC EQUATIONS

**FIGURE 1.** shows computational model used in the analysis. The computational domain is selected as 2.6 m×2.0 m×2.5 m in *x*, *y*, *z* directions. The height of breathing human is 1.6 m.

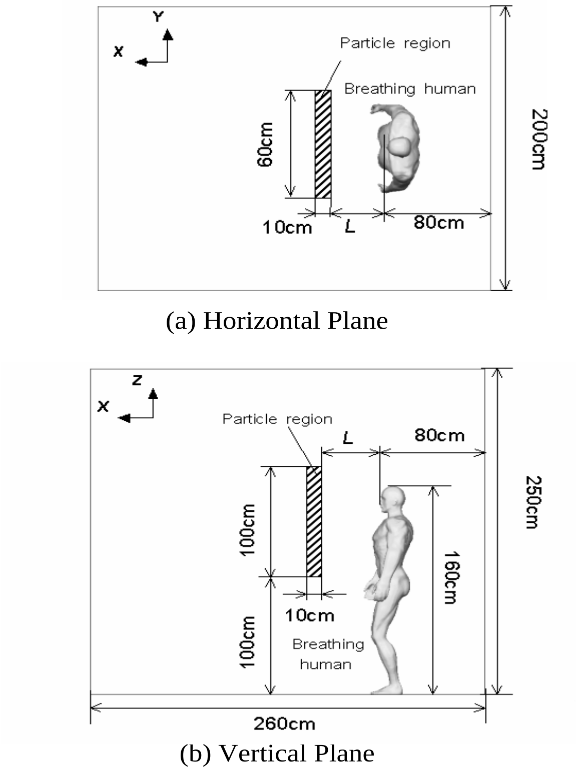

**FIGURE 1.** Computational Model

CP832, *Flow Dynamics, The Second International Conference on Flow Dynamics*
edited by M. Tokuyama and S. Maruyama
© 2006 American Institute of Physics 0-7354-0324-4/06/$23.00

The governing equations for flow are the mass conservation and the Navier-Stokes equations.

$$\nabla \cdot \vec{U} = 0 \quad (1)$$

$$\rho\left[\frac{\partial u}{\partial t} + \nabla \cdot \left(\vec{U}u\right)\right] = -\frac{\partial p}{\partial x} + \mu\nabla \cdot (\nabla u)$$

$$\rho\left[\frac{\partial v}{\partial t} + \nabla \cdot \left(\vec{U}v\right)\right] = -\frac{\partial p}{\partial y} + \mu\nabla \cdot (\nabla v) \quad (2)$$

$$\rho\left[\frac{\partial w}{\partial t} + \nabla \cdot \left(\vec{U}w\right)\right] = -\frac{\partial p}{\partial z} + \mu\nabla \cdot (\nabla w)$$

In these equations, $\rho$ is the density and stays constant in the simulations, $p$ is the static pressure, and $\mu$ is the viscosity. $\vec{U}$ is the velocity of the surrounding air. $u$, $v$, $w$ are the velocity of each component. As a turbulence model, standard $k$-$\varepsilon$ model was adopted. It involves solutions of transport equations for turbulent kinetic energy and its rate of dissipation. The model adopted in the simulation is based on Launder and Spalding [11] and is expressed as

$$\mu_t = \rho\frac{C_\mu k^2}{\varepsilon} \quad (3)$$

The transport equations for $k$ are

$$\rho\left[\frac{\partial k}{\partial t} + \nabla \cdot \left(\vec{U}k\right)\right] = \rho P - \rho\varepsilon + \nabla \cdot \left[\left(\mu + \frac{\mu_t}{\sigma_k}\right)\nabla \varepsilon\right] \quad (4)$$

And the transport equations for $\varepsilon$ are

$$\rho\left[\frac{\partial \varepsilon}{\partial t} + \nabla \cdot \left(\vec{U}\varepsilon\right)\right] = C_{\varepsilon_1}\frac{\rho P\varepsilon}{k} - C_{\varepsilon_2}\frac{\rho\varepsilon^2}{k} + \nabla \cdot \left[\left(\mu + \frac{\mu_t}{\sigma_\varepsilon}\right)\nabla \varepsilon\right] \quad (5)$$

The five constants used in this model are

$$C_\mu = 0.09,\ C_{\varepsilon_1} = 1.44,\ C_{\varepsilon_2} = 1.92$$
$$\sigma_k = 1.0,\ \sigma_\varepsilon = 1.3 \quad (6)$$

The motions of particulates were analyzed by solving the Lagrangian equations for each particle. The equation of particle motion is

$$m_\mathrm{p}\frac{d\vec{u}}{dt} = C_\mathrm{D}\rho\left(\vec{U} - \vec{u}\right)\left|\vec{U} - \vec{u}\right|\frac{A_\mathrm{p}}{2} + m_\mathrm{p}\vec{g} + \vec{F}_\mathrm{Saff} + \vec{F}_\mathrm{P} + \vec{F}_\mathrm{B} \quad (7)$$

where $m_\mathrm{p}$ is the mass of particle and $\vec{u}=(u, v, w)$ is its velocity vector, $C_\mathrm{D}$ is the drag coefficient, $\rho$ is the density of surrounding air, $A_\mathrm{p}$ is the projected area of a particle, and $\vec{g}$ is the gravity vector, respectively. For a spherical particle, $A_\mathrm{p}=\pi d^2/4$ where $d$ is the particle diameter. Each of the force considered in the analysis is Stokes drag with Cunningham correction [5], gravity, Saffman lifting force, pressure gradient force, and Brownian diffusion [12]. The following correlations are used to calculate the drag coefficient,

$$C_\mathrm{D} = \frac{1}{C_\mathrm{C}} \cdot \frac{24}{Re} \quad (for\ Re < 1)$$

$$C_\mathrm{D} = \frac{24}{Re}\left[1 + 0.15Re^{0.687}\right] \quad (for\ 1 < Re < 10^3) \quad (8)$$

For very small particles, when the size of particles is of the same magnitude as distance between gas molecules, slip occurs. Cunningham correction factor to fluid drag on particles should be applied for rarefied fluid-particle flows. Cunningham correction factor is

$$C_\mathrm{C} = 1 + Kn\left[1.227 + 0.42\exp\left(-\frac{0.85}{Kn}\right)\right] \quad (9)$$

The Knudsen number, $Kn$, may be evaluated based on molecular mean free path of the fluid and particle diameter as

$$Kn = \frac{2\lambda}{d} \quad (10)$$

Where $\lambda$ is the molecular mean free path. The Saffman lifting force arises due to the surface pressure distribution on a particle in the presence of a velocity gradient in the flow field and plays an important role in determining particle deposition patterns in simulations. Generalized Saffman lifting force can be expressed as

$$\vec{F}_\mathrm{saff} = 1.61d^2\sqrt{\mu\rho}\left|\vec{\omega}\right|^{-\frac{1}{2}}\left[\left(\vec{U} - \vec{u}\right) \times \vec{\omega}\right] \quad (11)$$
$$\vec{\omega} = \nabla \times \vec{U}$$

The pressure gradient force on a particle is given by

$$\vec{F}_\mathrm{P} = -\frac{\pi}{6}d^3\nabla p \quad (12)$$

where $\nabla p$ is the local pressure gradient. The Brownian diffusion experienced by particles results from the impact of carrier fluid molecules on the particles and is significant for sub-micron particles. Brownian diffusion can be evaluated as

$$\vec{F}_\mathrm{B} = \vec{R}\sqrt{\frac{216}{\pi}\frac{\mu k_B T}{C_\mathrm{C}\rho^2 d^5\Delta t}} \quad (13)$$

where $k_B$ is the Boltzmann constant, $\Delta t$ is the time step, and $\vec{R}$ is a Gaussian random number.

## CONDITIONS FOR NUMERICAL ANALYSIS

In three-dimensional numerical simulations, general-purpose software, CFD-ACE+2004 (CFD Research Corp.) was used. The grid configuration is shown in **FIGURE 2**. Linear orthogonal grid is mainly used in the region distant from the human body. Boundary-fitted grid is used near the human body. The maximum cell dimensions ranges from 1.1 mm to 325 mm. The total numbers of cell are 110077. The grids were generated by mesh generator CFD-VisCART (CFD Research Corp.). The finite volume approach is adopted, and then governing equations are numerically solved over each of control volumes. A co-located cell-centered variable arrangement is employed. The checkerboard instability is circumvented by Rhie and Chow [13]. SIMPLEC scheme has been adopted for flow phase analysis, and is an enhancement to the well known SIMPLE algorithm [14].

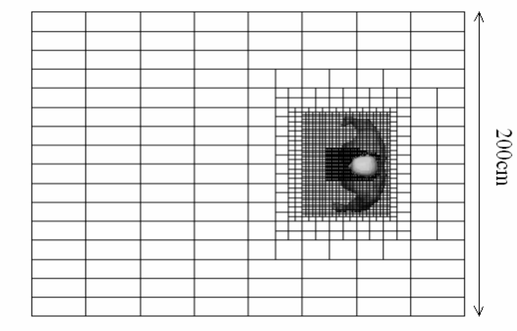

(a) Horizontal Plane

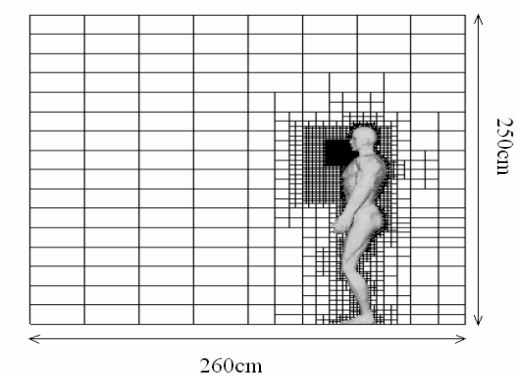

(b) Vertical Plane

**FIGURE 2.** Grid Configuration

The basic equations are numerically solved by the Euler method. The calculation conditions are as follows: The time step is selected 0.01 s, and 6000 steps are used. Residuals are small enough to deeming them convergence. It is assumed the unsteady flow rate in breathing is sinusoidal with a period of 4.0 s. This means 15 cycles of breathing are calculated. It is also assumed the human breathes only through his nose with uniform flow rate of 2.7 L/min. The area of his nostrils is 1.17 $cm^2$. The test particles size is 1 μm in diameter with density of 10 $kg/m^3$. These are typical values for bacteria and DEP.

The boundary conditions for the simulation are as follows: Whole areas of the front, the right, and the left surface against human body are set as velocity-given boundary and expressed as

$$\begin{aligned} &\overline{u} = \overline{v} = \overline{w} = 0 \\ &k = 2.88 \times 10^{-5} \quad \mathrm{m^2/s^2} \\ &\varepsilon = 2.13 \times 10^{-6} \quad \mathrm{m^2/s^3} \\ &T = 300 \quad \mathrm{K} \\ &P = 0 \end{aligned} \tag{14}$$

where $\overline{u}, \overline{v}, \overline{w}$ are the average values of each velocity component. And other surfaces including the human surface are set as

$$u = v = w = 0 \tag{15}$$

and the nose is given as

$$\begin{aligned} &u = f(t)\cos\alpha, v = 0, w = f(t)\sin\alpha \\ &k = 2.88 \times 10^{-5} \quad \mathrm{m^2/s^2} \\ &\varepsilon = 2.13 \times 10^{-6} \quad \mathrm{m^2/s^3} \\ &T = 300 \quad \mathrm{K} \\ &P = 0 \end{aligned} \tag{16}$$

where $\alpha$ is the breathing angle between the vertical lines and breathing velocity vector. Velocity is a time function *f (t)* and breathing volume is a product of multiplying *f (t)* by area of nostrils as **FIGURE 3**.

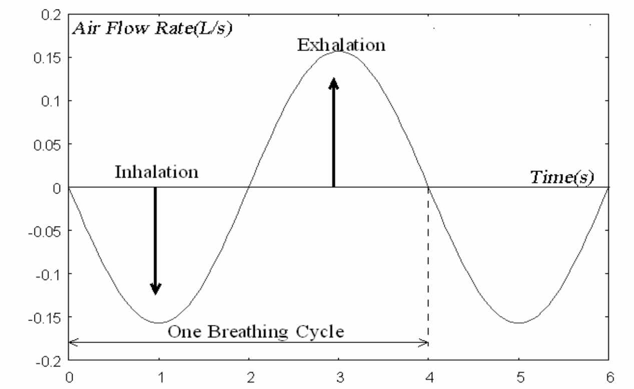

**FIGURE 3.** Breathing Process

As a pollutant source, a thousand particles are randomly placed in the box region (0.1 m×0.6 m×1.0 m). The particle region stay away *L* cm from the human nose, and we describe *L* as source distance. Particles are set for initial condition as

$$u_p = v_p = w_p = 0 \tag{17}$$

where $u_p$, $v_p$, $w_p$ are particle velocities. Particles are totally absorbed at the nose, on walls, and human surface. Initially, the fluid is considered as stationary fluid.

## RESULTS AND DISCUSSION

We first examined the dependence between breathing angles and flow field by a way of comparison of the two dimensional model. Breathing angles were set at 0, 30, 60, and 90 degree. *L* was 0.1 m at this time. The relationship among breathing angles and time to reach the nose and particle captured efficiency is shown in **TABLE 1.**

**TABLE 1.** Time to reach nose and particle captured efficiency.

| **Degree** <br> ° | **Time to reach nose** <br> s | **Captured efficiency** <br> % |
|---|---|---|
| Breathing angle=0 | 7 | 2.9 |
| Breathing angle=30 | 13 | 1.4 |
| Breathing angle=60 | Not Reach | 0 |
| Breathing angle=90 | Not Reach | 0 |

The particle captured efficiency tends to become higher and particles tend to reach the human nose faster as the breathing angle becomes smaller.

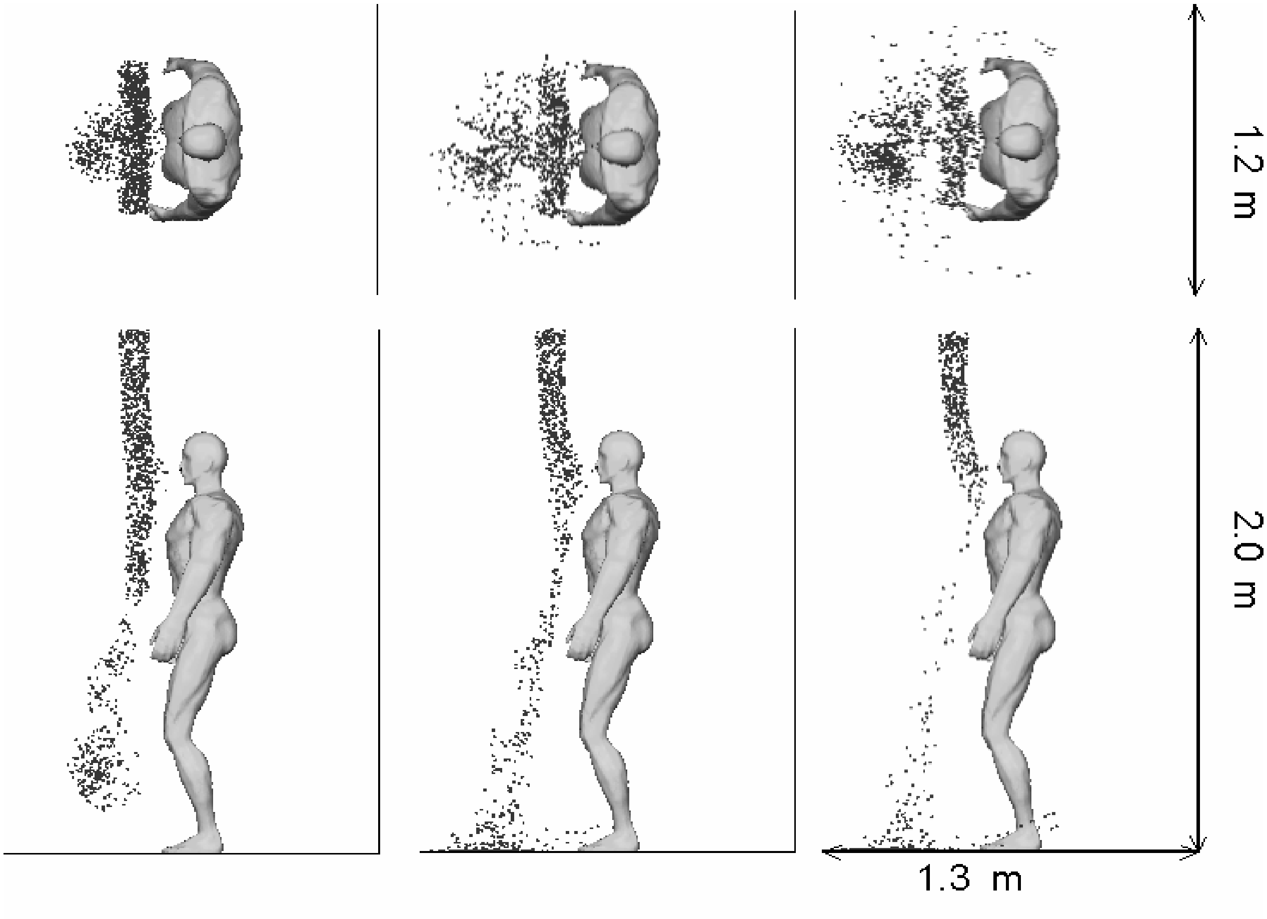

(a) *t*=15 s (b) *t*=30 s (c) *t*=45 s

**FIGURE 4.** Particles Distribution for Breathing Angle of 30 Degree

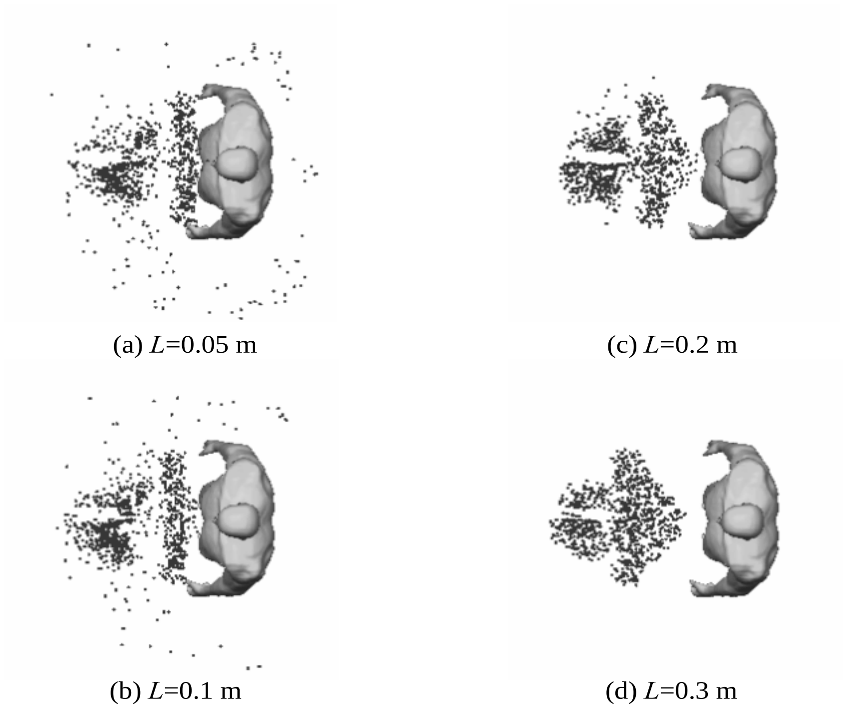

**FIGURE 5.** Particles Distribution for Various Source Distances from the Top after 60 s

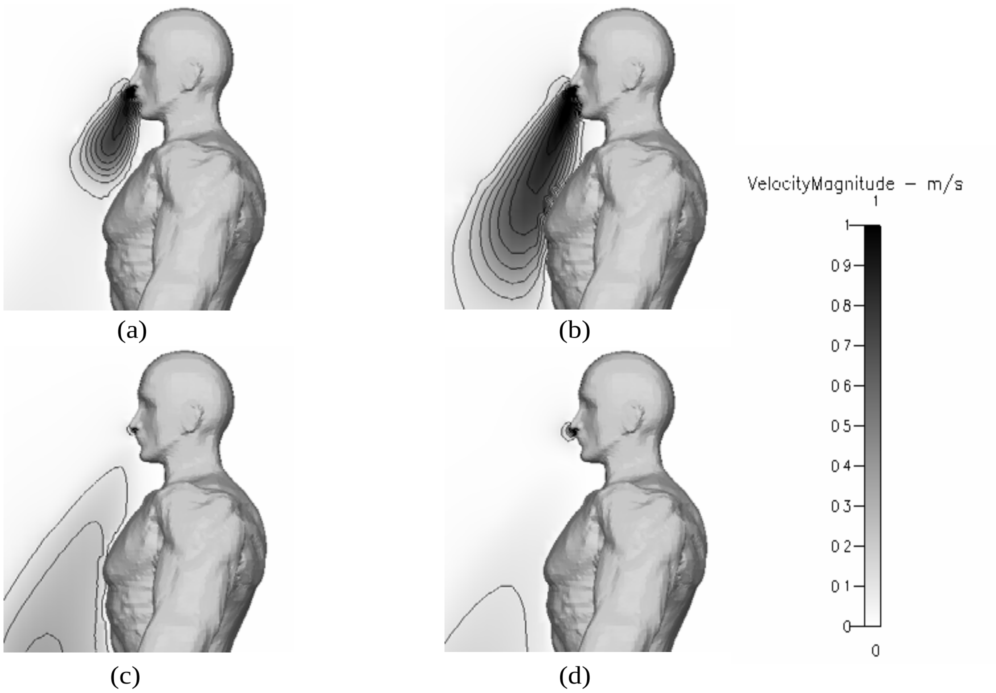

**FIGURE 6.** Velocity Distribution (a) $t$=0.8 s, (b) $t$=1.4 s in Exhalation and (c) $t$=0.4 s, (d) $t$=1.4 s in Inhalation

Particles reach the human in 7 s at the earliest, and the particle captured efficiency is 2.9% at the maximum, when breathing angle is 0 degree. No particles are captured with breathing angle of 60 degree or larger. **FIGURE 4. (a) — (c)** show the particles distribution for breathing angle of 30 degree after 15, 30, and 45 s of breathing. For breathing angles of 0 and 30 degree, particles move away from human body and right-left unsymmetrical vortexes are induced. For breathing angles of 60 and 90 degree, particles just move away and no vortexes are induced. Particles are drawn from upper region of the human. Thus vertical direction flow is a major factor for capturing particles. Breathing angles are important parameters as breathing angles influence z component of the flow.

We secondly examined the dependence of source distance. Source distances were set at 0.05, 0.1, 0.2, and 0.3 m. Breathing angle was 30 degree at this time. **TABLE 2.** show the result as a parameter of source distance. **FIGURE 5. (a) – (d)** show the particles distribution for various source distances. Particles reach the human when source distance ($L$) is 0.05, 0.1, and 0.2 m, while particles do not reach the human when $L$ is 0.3 m. This means the boundary separate captured from not-captured exist between $L$=0.2 m and $L$=0.3 m. Particles are less captured and reach slower toward increase of source distance. Particles can reach 0.6 m ahead and 1.0m wide for the maximum at L=0.05 m.

**TABLE 2.** Time to reach nose and particle captured efficiency.

| Source distance | Time to reach nose | Captured efficiency |
|---|---|---|
| m | s | % |
| $L$=0.05 | 9 | 2.6 |
| $L$=0.1 | 13 | 1.4 |
| $L$=0.2 | 24 | 0.1 |
| $L$=0.3 | Not Reach | 0 |

When we look at the flow field, exhalation has an impact on the field as shown in **FIGURE 6. (a) ― (d)**. In the early period of exhalation, the respiration region is small and the flow is complicated. The respiration area becomes larger and the velocity becomes higher as the time goes on. The downward stream caused by exhalation remains during inhalation, which is to say the downward stream is small in scale, but always stays and particles go down. Note that each breathing cycle seems self-dependence.

## CONCLUSIONS

We focus on the particulate pollutant in the air and three-dimensional numerical simulation was carried out. The effects of breathing angle and source distance ware clarified around human with unsteady breathing. The main results are summarized as follows:

(1) Exhalation has an impact on the flow field more than inhalation.

(2) The particle captured efficiency tends to become higher and particles tend to reach the human nose faster as source distance becomes near from human body. Particles reach the human nose when source distance is less than 0.2 m. No particles are reached beyond 0.3 m. The maximum 2.6% of particles are captured when the source distance is 0.05 m.

(3) The particle captured efficiency tends to become higher and particles tend to reach the human nose faster as the breathing angle becomes smaller. The human can inhale particles only breathing angle of 30 degree or smaller.

(4) Throughout all simulations, particles are drawn from upper region of the human. When particles are captured, motions of particles away from the human body with right-left unsymmetrical vortexes are observed. Otherwise, particles just move away and no vortexes are induced.
